# Applied Precision Cancer Medicine in Neuro-Oncology

**DOI:** 10.1038/s41598-019-56473-0

**Published:** 2019-12-27

**Authors:** H. Taghizadeh, L. Müllauer, J. Furtner, J. A. Hainfellner, C. Marosi, M. Preusser, G. W. Prager

**Affiliations:** 1 Department of Medicine I, Clinical Division of Oncology, Medical University of Vienna, Vienna, Austria; 2 Comprehensive Cancer Center, Vienna, Austria; 3 Clinical Institute of Pathology, Medical University Vienna, Vienna, Austria; 4 Department of Biomedical Imaging and Image-guided Therapy, Medical University of Vienna, Vienna, Austria; 5 Institute of Neurology, Medical University of Vienna, Vienna, Austria

**Keywords:** Cancer, Cancer genomics, CNS cancer

## Abstract

Brain tumours that are refractory to treatment have a poor prognosis and constitute a major challenge in offering effective treatment strategies. By targeting molecular alterations, precision cancer medicine may be a viable option for the treatment of brain tumours. In this retrospective analysis of our PCM platform, we describe the molecular profiling of primary brain tumours from 50 patients. Tumour samples of the patients were examined by a 161-gene next-generation sequencing panel, immunohistochemistry, and fluorescence *in situ* hybridization (FISH). We identified 103 molecular aberrations in 36 (72%) of the 50 patients. The predominant mutations were *TP53* (14.6%), *IDH1* (9.7%) and *PIK3CA* (6.8%). No mutations were detected in 14 (28%) of the 50 patients. IHC demonstrated frequent overexpression of EGFR and mTOR, in 38 (76%) and 35 (70%) patients, respectively. Overexpression of PDGFRa and PDGFRb were less common and detected in 16 and four patients, respectively. For 35 patients a targeted therapy was recommended. In our database, the majority of patients displayed mutations, against which targeted therapy could be offered. Based on our observations, PCM may be a feasible novel treatment approach in neuro-oncology.

## Introduction

Effective alternative therapies in adult patients with primary brain tumours relapsing after standard therapy is often lacking. For most neuro-oncological patients, there is no standard treatment recommendation after failure of first-line therapy. This is also true for patients with a glioblastoma, which constitutes a large proportion of patients treated by neuro-oncologists. Glioblastoma multiforme (GBM) is the most frequent malignant brain tumour, making up over 50% of all gliomas and 16% of all primary brain tumours^1^. In addition, for patients with relapsed diffuse or anaplastic astrocytomas or oligodendrogliomas, there are no standard alternative therapeutic recommendations, and for patients with rare brain tumours and for the few patients with relapsed or progressive meningiomas, further neurosurgical or radiotherapeutic treatment options may be very limited or absent.

The cancer database GLOBOCAN 2018 revealed that roughly 300,000 patients (1.6% of all new cancer cases) were diagnosed with a brain tumour, and 241,000 patients (2.5% of all cancer deaths) died of this disease^2^. According to GLOBOCAN 2018, brain tumours are the 19th most common cancer^2^. But despite their relatively low frequency, when compared with other malignant diseases, brain tumours cause a disproportionate amount of morbidity and mortality, partly because of the critical location of the tumour mass^3^. The management of advanced and relapsed brain tumours poses a great challenge to physicians and healthcare providers and requires co-operation between and management by a multidisciplinary team. There is no consensus on treatment pathways in recurrent or progressive brain tumours, and the existing therapeutic options are limited^4,5^.

In recent years, there has been an effort to progressively individualize therapy options in specific cancers. In a few particular cancers, treatment with immunotherapeutics or tyrosine kinase inhibitors, tailored to the individual, are possible, for example, trastuzumab in human epidermal growth factor receptor 2 (HER2)-positive breast cancer or gastric cancer, imatinib in philadelphia chromosome positive chronic myelogenous leukaemia (Ph + CML) or in *KIT*+ gastrointestinal stromal tumour (GIST), pazopanib and sunitinib in advanced renal cell carcinoma (RCC), or *BRAF*-directed therapy with vemurafenib or dabrafenib/trametinib in melanoma^6–8^.

Emerging techniques, such as profiling tumour molecular alterations and mutations, identifying molecular targets amenable to specific treatments, and developing drug treatments specific to an individual patient, provide the potential for novel and effective therapies. The ground-breaking pilot trial by Daniel von Hoff have ushered in a new era of medicine. This approach is known by a number of different names, including individualized, stratified, tailored or precision cancer medicine (PCM)^9^. The main rationale of PCM is to match a therapeutic agent to its corresponding molecular target, to allow a precise treatment tailored to a specific patient. It aims to achieve a better and more sustained response than more generic treatments, without damaging healthy cells and tissues^10^. We conducted a retrospective subgroup analysis of all 50 patients with primary brain tumour (PBT) that had been enrolled and profiled in our special PCM platform of molecular oncological diagnostics and therapy (MONDTI) of the comprehensive cancer centre of the Medical University of Vienna (CCC-MUV). We sought to map the molecular profiles (MP) of highly advanced, mainly relapsed PBT and to specifically target the found molecular alterations.

## Materials and Methods

### Patients and design of the precision medicine platform

Patients with progressive and mainly recurrent PBT who had progressed to all standard treatment options were eligible for inclusion in MONDTI, provided archival tissue samples were available. Patients had to have an Eastern Cooperative Oncology Group (ECOG) performance status of 0 or 1. MONDTI is not a clinical trial, but intends to provide the possibility of a targeted therapy to patients where no active anti-tumoural treatment is available.

The study was performed according to the International Conference on Harmonization E6 requirements for Good Clinical Practice and with the ethical principles described in the Declaration of Helsinki. Patients had to provide informed consent before inclusion in MONDTI. Furthermore, the Institutional Ethics Committee of the Medical University of Vienna has also approved this subanalysis (Nr. 1039/2017).

### Tissue samples

Formalin-fixed, paraffin-embedded tissue sections from patients with advanced PBT that were resistant to all standard therapy options were sent to or retrieved from the archive of the Department of Neuropathology and sent to the Department of Pathology, Medical University Vienna, Vienna, Austria.

### Cancer gene panel sequencing

DNA was obtained from paraffin-embedded tumour tissue blocks with a QIAamp Tissue KitTM (Qiagen, Hilden, Germany), and 10 ng DNA per sample was employed for sequencing. The DNA library was generated by multiplex polymerase chain reaction with the 161-gene next-generation sequencing panel of Oncomine Comprehensive Assay v3 (Thermo Fisher Scientific, Waltham, MA, USA).

### Immunohistochemistry (IHC)

IHC was done utilizing 2-μm-thin tissue sections. The following antibodies were used: ALK (clone 1A4; Zytomed, Berlin, Germany), cluster of differentiation 30 (CD30) (clone BerH2; Dako, Vienna, Austria), CD20 (clone L26; Dako), epidermal growth factor receptor (EGFR) (clone 3C6; Ventana), oestrogen receptor (ER) (clone SP1; Ventana), HER2 (clone 4B5; Ventana), HER3 (clone SP71; Abcam), KIT (clone 9.7; Ventana), MET (clone SP44; Ventana), phosphorylated- mechanistic target of rapamycin (p-mTOR) (clone 49F9; Cell Signaling Technology, Danvers, MA, USA), platelet-derived growth factor receptor (PDGFRA) (rabbit polyclonal; Thermo Fisher Scientific), PDGFRB (clone 28E1; Cell Signaling Technology), programmed death-ligand 1 (PD-L1) (clone E1L3N; Cell Signaling Technology), progesterone receptor (PR) (clone 1E2; Ventana), phosphatase and tensin homolog (PTEN) (clone Y184; Abcam), and ROS1 (clone D4D6; Cell Signaling Technology). An immunohistochemical score was calculated by multiplying the percentage of positive cells with their corresponding staining intensity (0 = negative, 1 = weak, 2 = moderate, 3 = strong), as described here: (maximum 300) = (% negative × 0) + (% weak × 1) + (% moderate × 2) + (% strong × 3).

The cut offs employed for each immunohistochemical stain used for treatment recommendation as follows: Oestrogen receptor and progesteron receptor: ≥10% positive tumour cells of any staining intensity, HER2: positive, Score 2+ or 3+ and additionally presence of an HER2 gene amplification with an HER2:centromer 17 ratio ≥2, KIT: IHC score ≥100 and presence of a pathogenic/likely pathogenic KIT mutation, p-mTOR: IHC score ≥100 and loss of PTEN expression or presence of a pathogenic/likely pathogenic PTEN mutation, PDGFRA and PDGFRB: IHC score of ≥100, PD-L1: tumour proportion score ≥1.

### Fluorescence *in situ* hybridization

FISH was done with 4-μm-thick formalin-fixed, paraffin-embedded tissue samples. The following fluorescent probes were utilized: ALK (2p23.1; Abbott, Abbott Park, IL, USA), RET (10q11; Kreatech, Berlin, Germany), PTEN (10q23.31)/Centromere 10, and ROS1 (ZytoVision, Bremerhaven, Germany). Two hundred cell nuclei per tumour were assessed. The cut-off level for an aberrant ALK, RET, and ROS1 FISH was ≥15% of cells with a split-apart signal. The PTEN FISH was considered positive for PTEN gene loss with ≥30% of cells with only one or no PTEN signals. A chromosome 10 centromere FISH probe served as a control for ploidy of chromosome 10.

### Multidisciplinary boards (molecular tumour boards for PCM)

After thorough examination of the molecular profile of each tumour sample by a qualified and competent molecular pathologist, the results and findings were reviewed in a multidisciplinary tumour boards (MTB) that were held every other week. Members of the board included molecular pathologists, radiologists, clinical oncologists, biostatisticians, and basic scientists. The MTB recommended the targeted therapy based on the specific molecular profile of each patient. The targeted therapies included tyrosine kinase inhibitors, checkpoint inhibitors (e.g. anti- PD-L1 monoclonal antibodies), and growth factor receptor antibodies with or without endocrine therapy. The treatment recommendations by the MTB were prioritized dependent on the level of evidence from high to low according to phase III to phase I trials.

In cases where more than one druggable molecular aberration was identified, the MTB recommended a therapy regimen to target as many molecular aberrations as possible, with special consideration to toxicity profile of each antitumoural agent and their potential interactions. Since all patients were given all available standard treatment options for their cancer disease prior to their inclusion in our PCM platform, nearly all targeted agents were suggested as off-label use. If the tumour profile and the clinical characteristics of a patient met the requirements of a clinical trial for targeted therapies that was conducted in our cancer centre, patients were preferentially asked if they wanted to participate in this trial.

### Descriptive statistics

For data description, we used measures of central tendency including the mean and median. We also used the method of frequency distribution to delineate the characteristics of the PBT patients.

## Results

Fifty patients diagnosed with a primary brain tumour were included in this subgroup analysis from the cohort of the PCM project MONDTI, that has so far profiled 550 patients with various highly advanced cancer types. In this analysis, all patients were Caucasians. There were 26 men and 24 women, diagnosed with a total of 24 different types of PBT. The median age at first diagnosis was 39 years, range 10 to 71, and the median age at the time when the molecular profiling was performed was 45 years, range 10 to 72 (Table 1). The tumour tissue was obtained during surgical intervention.

**Table 1:** Patient characteristics (N = 50).

Patient Characteristics	Number
Mean Age at Fist Diagnosis	39
Mean Age at molecular profiling	45
Men	24
Women	26
Caucasian	50
Types of PBT	24
Relapsed disease	42
Received therapy lines	2 (1–4)
Brain tumour types	25

Thirty patients had a diagnosis of glioma. The analysis also included rare brain tumours, including papillary tumour of the pineal region (PTPR), pineal parenchymal tumour of intermediate differentiation (PPTID), gliofibroma, and a very rare, as yet unclassified, type of glioma with loss of H3 K27me3 and absence of *H3 K27* mutation that has been verified by IHC and sequencing, and described only in extremely rare cases.

There was a time span of between six months and two years between diagnosis and when molecular profiling was performed. By the time of molecular profiling, 42 patients had suffered relapses of their malignant disease and had received a median of two courses of drug therapy (range 1–4). All patients had undergone at least one surgical intervention.

In total, we identified 103 molecular aberrations in 36 patients. The predominant mutations were *TP53* (14.6%), *IDH1* (9.7%) and *PIK3CA* (6.8%). No mutations were detected in 14 patients. Some patients were found to have more than one mutation. The three patients diagnosed with an anaplastic oligodendroglioma had a total of 37 mutations. In contrast, ten patients with GBM were found to have a total of 27 aberrations. In four of the 30 patients with glioma, the MGMT promoter was methylated. *IDH1* was mutated in ten patients. *IDH2* was mutated in one patient with secondary GBM. Interestingly, *BRCA1* was mutated in one patient diagnosed with GBM, and *BRCA2* mutation was shown in one sample of anaplastic oligodendroglioma. A genetic alteration of *EGFR* was observed in three patients; however, none of them was EGFRvIII-positive. See Tables 2 and 3 for a detailed analysis of all the reported mutations. Apart from *TP53, IDH1, PIK3CA, ATM, BRAF and CDKN2A*, mutations in other genes were detected in only one to two cases each.

**Table 2:** Types of primary brain tumours, genetic alterations and recommended therapy.

Brain tumour type according to WHO classification of 2016	Number	WHO Grade	Number of detected mutations and MGMT promoter methylation	Number of therapy recommendations
Glioblastoma multiforme (GBM)	10	IV°	*6xTP53*, *2xCREBBP, 2xPIK3CA*, *1xARID1A, 1x BAP1, 1x BRAF, 1xBRCA1*, *1xEGFR, 1xIDH1, 1x IDH2, 1xKDR, 1x NOTCH1, 1x PIK3CB, 1xPIK3R1, 1xPOLE*, *1x PTEN, 1x RB1, 1xSMAD4, 1x SMO, 1x STK11;* 2xMGMT promoter methylation	EGFR inhibitor (n = 2), imatinib (n = 4), pembrolizumab (n = 2)
Anaplastic Hemangiopericytoma	4	III°	*1xFGFR4, 1xPOLE*	FGFR inhibitor (n = 1), sunitinib (n = 1)
Anaplastic Oligoastrocytoma	3	III°	*2xIDH1, 1xPDGFRA*	Erlotinib (n = 1), imatinib (n = 1)
Anaplastic Astrocytoma	3	III°	*1xIDH1, 1xPTPN11, 1xTP53*	Erlotinib (n = 1), everolimus (n = 1), imatinib (n = 1)
Anaplastic Oligodendroglioma, IDH-mutant, 1p19q co-deleted	3	III°	*3xIDH1, 2xMLH1, 2xATM, 1xABL, 1xALK, 1xAPC, 1xATR*, *1xBRAF, 1xBRCA2, 1xCCND3, 1xCDKN1B, 1xCDKN2A, 1xEGFR, 1xERBB4, 1xFGFR3*, *1xFGFR4, 1xIGF1R, 1xMRE11A, 1xMTOR, 1xNOTCH1, 1xPIK3CA, 1xPIK3R1, 1xPOLE, 1xPTCH, 1xPTPN11, 1xRAD51C, 1xRB*, *1xSLX4, 1xSMAD4, 1xSMARCB1, 1xSTK11, 1x TSC2, 1xTP53* 1xMGMT promoter methylation	Imatinib (n = 1), pembrolizumab (n = 1), sunitinib (n = 1)
Anaplastic pleomorphic xanthoastrocytoma (PXA)	2	III°	*1xBRAF V600E;* 1xMGMT promoter methylation	Dabrafenib and trametinib (n = 1), imatinib (n = 1)
Oligoastrocytoma	2	II°	*2xIDH1, 2xTP53, 1xATM*	Olaparib (n = 1), imatinib (n = 1)
Atypical meningioma	3	II°	*1xAKT1, 1xCDKN2A, 1xEGFR, 1xFGFR1, 1xMAF, 1xRET, 1xTP53*	Cetuximab (n = 1), imatinib (n = 1), AKT inhibitor (n = 1)
Medulloblastoma	2	IV°	*1xATR, 1xPIK3CA, 1xSMO, 1xTERT*	Vismodegib (n = 1)
Anaplastic ependymoma	2	III°	*1xATM*	Pembrolizumab (n = 1)
Chordoma	2	—	0	Imatinib (n = 1)
Hemangioblastoma	2	I°	*1xVHL*	Pazopanib (n = 1), PDGFRa inhibitor (n = 1)
Papillary tumour of the pineal region (PTPR)	1	III°	0	Sunitinib (n = 1)
Pituitary carcinoma – malignant prolactinoma	1	—	0	Pembrolizumab (n = 1)
Pilocytic astrocytoma	1	I°	*1xPIK3CA*	0
Melanotic schwannoma	1	I°	*1xGNAQ*	0
Myxopapillary ependymoma	1	I°	*1xKDR*	Sunitinib (n = 1)
Atypical choroid plexus papilloma (aCPP)	1	II°	*1xTP53*	0
Pineal parenchymal tumour of intermediate differentiation (PPTID)	1	III°	0	0
Diffuse midline glioma H3 K27M-mutant	1	IV°	*1xNF1* *1xTP53*	Imatinib (n = 1)
MPNST	1	—	0	Sunitinib (n = 1)
Diffuse astrocytoma	1	II°	*1xIDH1, 1xTP53*	0
Glioma with loss of H3 K27me3 and absence of H3 K27 mutation (described in rare cases)	1	—	*1xCDKN2A, 1xPIK3CA, 1xTP53*	0
Gliofibroma (described in rare cases)	1	—	*1xPIK3CA*	0

**Table 3:** Number of detected mutations.

Mutation	Number	Percent
*ABL1*	1	1.0%
*AKT1*	1	1.0%
*ALK*	1	1.0%
*APC*	1	1.0%
*ARID1A*	1	1.0%
*ATM*	4	3.9%
*ATR*	2	1.9%
*BAP1*	1	1.0%
*BRAF*	3	2.9%
*BRCA1*	1	1.0%
*BRCA2*	1	1.0%
*CCND3*	1	1.0%
*CDKN1B*	1	1.0%
*CDKN2A*	3	2.9%
*CREBBP*	2	1.9%
*EGFR*	3	2.9%
*ERBB4*	1	1.0%
*FGFR1*	1	1.0%
*FGFR3*	1	1.0%
*FGFR4*	2	1.9%
*GNAQ*	1	1.0%
*IDH1*	10	9.7%
*IGF1R*	1	1.0%
*IDH2*	1	1.0%
*KDR*	2	1.9%
*MAF*	1	1.0%
*MLH1*	2	1.9%
*MRE11A*	1	1.0%
*MTOR*	1	1.0%
*NF1*	1	1.0%
*NOTCH1*	2	1.9%
*PDGFRA*	1	1.0%
*PIK3CA*	7	6.8%
*PIK3CB*	1	1.0%
*PIK3R1*	2	1.9%
*POLE*	3	2.9%
*PTCH*	1	1.0%
*PTEN*	1	1.0%
*PTPN11*	2	1.9%
*RAD51C*	1	1.0%
*RB1*	2	1.9%
*RET*	1	1.0%
*SLX4*	1	1.0%
*SMAD4*	2	1.9%
*SMARCB1*	1	1.0%
*SMO*	2	1.9%
*STK11*	2	1.9%
*TERT*	1	1.0%
*TP53*	15	14.6%
*TSC2*	1	1.0%
*VHL*	1	1.0%
Total	103	100%

IHC detected EGFR overexpression in 38 patients, with a high median score of 180. Seventeen patients had an EGFR score between 200 and 300. The overexpression of mTOR was detected in 35 patients, and was lower than was EGFR overexpression, with a median score of the former of 65. PTEN expression was revealed in exactly half of the patients, with a median score of 100. Less common and of lower levels were overexpression of PDGFRa and PDGFRb, which were found in 15 and eight patients, respectively. Ten patients with PDGFRa overexpression and two patients with PDGFRb had a high-grade glioma, including GBM, anaplastic oligoastrocytoma, anaplastic oligodendroglioma and anaplastic pleomorphic xanthoastrocytoma (PXA). Tumour expression of PD-L1 was seen in six tumour samples, including two cases of GBM, and in one case each of anaplastic oligodendroglioma, malignant prolactinoma, anaplastic oligodendroglioma and anaplastic hemangiopericytoma. The progesterone receptor was expressed in five glioma patients, and expression of the oestrogen receptor was found in only one woman, who had a malignant prolactinoma. Overexpression of KIT was reported in four patients. Three of these patients had a concomitant PDGFRa overexpression. FISH analysis revealed a loss of PTEN in three patients.

In over two-thirds (35) of the 50 patients, a targeted therapy was suggested, based on the identified genetic mutations. The most frequently recommended specific treatments were imatinib (n = 12), sunitinib (n = 5) and pembrolizumab (n = 5). For five patients, a more general treatment recommendation was produced: EGFR inhibitor (n = 2), AKT inhibitor (n = 1), FGFR inhibitor (n = 1), PDGFRa inhibitor (n = 1). For two patients, erlotinib was considered. Everolimus, cetuximab, dabrafenib/trametinib, olaparib, pazopanib and vismodegib were each proposed in one case each. Table 4 describes the rationale behind the specific therapy suggestions. In two cases, sunitinib or erlotinib were recommended, after the patients had received a course of imatinib but had subsequently become refractory to imatinib treatment. Except for two patients, all patients with GBM (n = 8) were given a therapy suggestion. For all patients with anaplastic oligodendroglioma, anaplastic astrocytoma, meningioma, oligoastrocytoma or PXA, an experimental therapy option was suggested.

**Table 4:** Rational for therapy recommendations.

Therapeutic agent (trading name)	Targets	Overview of current FDA approval in different entities	Overview of current EMA approval in different entities
Imatinib (Gleevec)	PDGFR, KIT	Ph + CML, KIT + GIST, MDS/MPD associated with PDGFR, Ph + ALL	Ph + CML, KIT + GIST, MDS/MPD associated with PDGFR, Ph + ALL
Sunitinib (Sutent)	PDGFR, KIT, VEGFR, RET, GCSFR, FLT3	RCC, PDAC, GIST	RCC, PDAC, GIST
Pazopanib (Votrient)	KIT, FGFR, PDGFR, VEGFR	RCC, soft tissue sarcoma	RCC, soft tissue sarcoma
Erlotinib (Tarceva)	EGFR	NSCLC, PDAC	NSCLC, PDAC
Pembrolizumab (Keytruda)	PD-1, hypermutability,	Melanoma, NSCLC, HNSCC, HL, Urothelial carcinoma, microsatellite instability-high cancer, gastric cancer, cervical cancer	Melanoma, NSCLC, HNSCC, HL, Urothelial carcinoma
Cetuximab (Erbitux)	EGFR expression	CRC, HNSCC	CRC, HNSCC
Everolimus (Afinitor)	mTOR expression	Breast cancer, PNET, RCC, Renal angiomyolipoma, subependymal giant cell astrocytomas (SEGAs) in with tuberous sclerosis complex (TSC)	Breast cancer, RCC, Neuroendocrine tumours of pancreatic, gastrointestinal or lung origin
Olaparib (Lynparza)	*BRCA1/2, ATM, CHEK2, PALB2*	Ovarian cancer, Breast cancer	Ovarian, fallopian tube or primary peritoneal cancer
Vismodegib (Erivedge)	*SMO*	Basal cell carcinoma	Basal cell carcinoma
Dabrafenib/Trametinib (Tafinlar/Mekinist)	*BRAF V600E*	BRAF V600E melanoma or NSCLC	BRAF V600E melanoma or NSCLC

## Discussion

In this report, we show that the information of individual genomic alterations in patients with primary brain tumours that are refractory to standard treatment has been translated into specific therapeutic recommendations. Notably in this study, the histological type was diverse, and patients with rare brain tumours were also included and investigated.

In this retrospective single-centre analysis, we present the molecular profiling (MP) of all 50 patients with PBT from the MONDTI cohort. Their disease was therapy-refractory and highly advanced. Tumour tissue was obtained from all patients and characterized for their MP. Subsequently, the genomic information of the patients was discussed in a multidisciplinary tumour board (MTB) for PCM to evaluate the possibility of a genomic-based treatment that was independent of the tumour’s histological classification (tissue-agnostic treatment). A treatment recommendation was derived for 35 patients from the MTB. The drugs were carefully selected to form an individualized treatment, taking into account the patient’s clinical and treatment history and concomitant therapies and comorbidities.

Tumour samples commonly displayed mutations in *TP53, IDH1 and PIK3CA*, and revealed a high median EGFR score. Common therapeutic agents recommended were imatinib, sunitinib and pembrolizumab. The detected mutations and IHC scores observed in gliomas in this analysis are in keeping with previous studies^11–13^. The analysis presented in this study shows that molecular profiling from tumour samples of patients and subsequent identification of therapeutic options with highly advanced PBT appears feasible and safe, and consistent with previous work. While outcome data are not presented in this study, previous work has shown that the drug treatments used here may be effective in improving clinical outcomes.

A similar study used molecular profiling to offer an individualized therapy only in patients with recurrent or advanced glioblastoma. They enrolled a limited number of 20 patients of whom seven actually received the recommended treatment. The investigators reported the feasibility of treatment plan guided by the identified molecular profiling^14^.

A pilot trial evaluated the feasibility and potential of precision medicine in young patients below 25 years with newly diagnosed diffuse intrinsic pontine glioma. Based on whole exome sequencing and RNA sequencing, a personalized therapy concept was developed for 15 patients^15^.

Another trial performed exome and transcriptome sequencing in 91 young patients with therapy refractory or relapsed cancer and integrated and incorporated the information into clinical management. In roughly half of the patients (46%), druggable molecular alterations were found and were considered in the treatment plan^16^.

Imatinib and sunitinib were offered in this current study as a treatment when overexpression of PDGFRa/b or KIT was identified. Haberler *et al*. have shown previously that, in a cohort of 101 glioblastoma patients, the expression of anti-PDGFRa and b, c-kit, c-abl and arg protein that can be targeted by imatinib^17^. According to a phase II trial in patients with recurrent gliomas, treatment with imatinib is well tolerated, with a limited antitumour activity^18^. Hassler *et al*. also tested the antitumour activity of imatinib in 24 recurrent glioblastoma patients with at least one tyrosine kinase expression that can be targeted by imatinib. Six patients survived over one year, and twelve patients achieved stable disease (SD). Median progression-free survival was 3 months and median overall survival was 6 months^19^. Based on preclinical data, sunitinib appears to impede brain tumour progression and reduce tumour-induced neurodegeneration in the microenvironment^20^. In this study, imatinib was preferred over sunitinib or erlotinib due to its relatively favourable and manageable safety profile and longer experience of its use.

Pazopanib has previously been used in a patient with hemangioblastoma and von Hippel-Lindau (VHL) disease. An important study published in The Lancet investigated the efficacy of pazopanib in 31 patients with VHL and reported an objective response in 13 patients (42%). However, treatment-related serious adverse events included one case each of appendicitis and gastritis, and one patient had a fatal central nervous system bleeding^21^.

Notably, we identified a *BRCA1/2* and an *ATM* mutation that are rational targets for Poly [ADP-ribose] polymerase 1 (PARP) inhibitors, for example olaparib^22^. It has been shown that olaparib penetrates the blood-brain barrier (BBB) and therefore easily accesses the tumour site, and it may enhance the cytotoxic effects of ionising radiation and temozolomide. Halford *et al*. treated 35 patients with olaparib and temozolomide and achieved six-month progression-free survival (PFS) rates in all patients, with a favourable safety profile^23^.

*BRAF V600E* mutation has been reported in a young woman with anaplastic PXA, who was offered dabrafenib/trametinib as tailored therapy. This drug combination has achieved Food and Drug Administration (FDA) approval for the treatment of melanoma displaying the *BRAF V600E* mutation. Our observation is in line with a retrospective study by Burger *et al*., who noted SD in three patients with recurrent, *BRAF V600E*-mutated malignant glioma after receiving dabrafenib^24^.

Pembrolizumab has been suggested in patients with tumour expression of PD-L1 or with a hypermutability. In a small study of 25 patients with refractory high-grade glioma, pembrolizumab was administered as salvage treatment and achieved a median overall survival of four months^25^. In May 2017, the FDA granted accelerated approval to pembrolizumab for the first tissue-agnostic indication in patients with unresectable or metastatic, microsatellite instability-high (MSI-H) or mismatch repair-deficient (dMMR) solid tumours that have progressed following prior treatment and which have no satisfactory alternative treatment options^26^.

For one male patient with recurrent medulloblastoma and *SMO* mutation, vismodegib was considered as targeted therapy in our analysis. The antitumour activity of vismodegib acting as a sonic hedgehog (SHH) pathway inhibitor has previously been confirmed by Robinson *et al*.^27^.

In patients with chordoma, PTPR and diffuse midline glioma imatinib or sunitinib were recommended due to expression of PDGFR^28,29^.

Despite the efforts and the investigated agents in brain tumours, progressive recurrent PBT have a very poor prognosis and there are only few FDA-approved therapeutic agents for brain tumours currently recommended or in use.

It is a difficult and complex task to classify and prioritize the plethora of reported genetic alterations and epigenetic changes of brain tumours, in order to identify molecular targets and to choose adequate tailored therapeutic measures. Most of the identified alterations are therefore currently undefined regarding their role in the pathogenesis of brain tumours and their therapeutic consequences and implications^30^.

With the exception of *TP53, IDH1, PIK3CA, ATM, BRAF and CDKN2A*, mutations in all other genes were detected only one to two times in the current study. This finding is consistent with the well-described extreme and complex intratumoural heterogeneity which occurs within the same tumour tissue; vascularization, proliferation, tumour mutational burden (TMB) and subclones are all known to be highly variable. The pattern of genetic and epigenetic aberrations changes both spatially and temporally. The tumour biology at metastatic sites is different from the primary site and differs again at the time point of relapse. In addition, it is known that the therapy itself can influence and inform the clonal tumour evolution, by creating new driver mutations in subclones that become insensitive to drugs^30,31^.

Another relevant issue is the drug delivery to the brain tumour. Tailored drugs applied in neuro-oncology must be capable of bypassing the physiological constraint of the BBB to reach the tumour^32^.

Taken together, the extremely complex tumour biology, the spatial and temporal heterogeneity in PBT genetics, and the difficulty of bypassing the BBB pose unique challenges for the management of brain tumours and in drug development. Further research is required to better comprehend the tumour biology.

One limitation of this subgroup analysis is that neurotrophin receptor kinases (*TRK*) fusion genes were not assessed and evaluated. TRK fusion genes are important oncogenic drivers in high-grade glioma that have a therapeutic consequence, as they can be targeted by specific inhibitors such as larotrectinib or entrectinib^33,34^. Another limitation is that the tumour tissue was obtained during surgical intervention and there was subsequently a time delay of between six months and two years until molecular profiling was performed. This limitation is important, since the tumour biology differs at the time of relapse or progression following the failure of initial therapy. Thus, the antitumour activity of targeted agents that are aimed at mutations present at the time of diagnosis may be reduced due to the changed molecular landscape of the tumour. For future analysis and studies, it would be of major clinical relevance to obtain genetic information of the tumour at the time of relapse or progression e.g. via liquid biopsy of cerebrospinal fluid to recommend the targeted therapy according to the current molecular characteristics of the tumour^35–38^.

Despite the limited number of patients included in this subgroup analysis, the results are promising. PCM clearly has the potential to inform treatment of PBT by expanding the therapeutic repertoire. Currently, PCM in neuro-oncology is in its infancy but has the potential to become more widely used in cancer drug development and therapy planning and strategy^11,31^.

## Supplementary information

Supplementary information
