# Supplementary material for: Applied Precision Cancer Medicine in Neuro-Oncology
Source: Sci Rep. 2019 Dec 27;9:20139. doi: 10.1038/s41598-019-56473-0 (PMC6934769; doi:10.1038/s41598-019-56473-0)
Supplement: Supplementary file 1 — Supplementary information [file 41598_2019_56473_MOESM1_ESM.docx]

**Applied Precision Cancer Medicine in Neuro-Oncology**

Taghizadeh H^1,2^, Müllauer L^3^, Furtner J^2,4^, Hainfellner JA^2,5^, Marosi C^1,2^, Preusser M^1,2^, Prager GW^1,2^

1 Department of Medicine I, Clinical Division of Oncology, Medical University of Vienna, Vienna, Austria

2 Comprehensive Cancer Center, Vienna, Austria

3 Clinical Institute of Pathology, Medical University Vienna, Vienna, Austria

4 Department of Biomedical Imaging and Image-guided Therapy, Medical University of Vienna, Vienna, Austria.

5 Institute of Neurology, Medical University of Vienna, Vienna, Austria

**Corresponding Author:**

Professor Gerald W Prager

Department of Medicine I

Clinical Division of Oncology

Comprehensive Cancer Center Vienna

Precision Cancer Medicine Unit

Medical University of Vienna

Email: [gerald.prager@meduniwien.ac.at](mailto:gerald.prager@meduniwien.ac.at)

Tel: 0043 1 40400-44500

List of gene targets in Oncomine Comprehensive Assay v3 (Thermo Fisher Scientific, Waltham, MA, USA) – 161 genes

| Hotspot genes | Full-length genes | Copy number genes | Gene fusions (inter- and intragenic) |
| --- | --- | --- | --- |
| AKT1  ALK  AR  ARAF  BRAF  BTK  CBL  CDK4  CHEK2  CSF1R  CTNNB1  DDR2  EGFR  ERBB2  ERB83  ERBB4  ESR1  EZH2  FGFR1  FGFR2  FGFR3  FLT3  FOXL2  GATA2  GNA11  GNAQ  GNAS  HNF1A  HRAS  IDH1  IDH2  JAK1  JAK2  JAK3  KDR  KIT  KNSTRN  KRAS  MAGOH  MAP2K1  MAP2K2  MAPK1  MAX  MED12  MET  MTOR  MYD88  NFE2L2  NRAS  PDGFRA  PIK3CA  PPP2R1A  PTPN11  RAC1  RAF1  RET  RHEB  RHOA  SF3B1  SMO  SPOP  SRC  STAT3  U2AF1  XPO1  AKT2  AKT3  AXL  CCND1  CDK6  ERCC2  FGFR4  H3F3A  HIST1H3B  MAP2K4  MDM4  MYC  MYCN  NTRK1  NTRK2  PDGFRB  PIK3CB  ROS1  SMAD4  TERT  TOP1 | ATM  BAP1  BRCA1  BRCA2  CDKN2A  FBXW7  MSH2  NF1  NF2  NOTCH1  PIK3R1  PTCH1  PTEN  RB1  SMARCB1  STK11  TP53  TSC1  TSC2  ARID1A  ATR  ATRX  CDK12  CDKN1B  CDKN2B  CHEK1  CREBBP  FANCA  FANCD2  FANCI  MLH1  MRE11A  MSH6  NBN  NOTCH2  NOTCH3  PALB2  PMS2  POLE  RAD50  RAD51  RAD51B  RAD51C  RAD51D  RNF43  SETD2  SLX4  SMARCA4 | AKT1  AR  CCND1  CCNE1  CDK4  CDK6  EGFR  ERBB2  FGFR1  FGFR2  FGFR3  FGFR4  FLT3  IGF1R  KIT  KRAS  MDM2  MDM4  MET  MYC  MYCL  MYCN  PDGFRA  PIK3CA  PPARG  TERT  AKT2  AKT3  ALK  AXL  BRAF  CCND2  CCND3  CDK2  CDKN2A  CDKN2B  ESR1  FGF19  FGF3  NTRK1  NTRK2  NTRK3  PDGFRB  PIK3CB  RICTOR  TSC1  TSC2 | ALK  AXL  BRAF  EGFR  ERBB2  ERG  ETV1  ETV4  ETV5  FGFR1  FGFR2  FGFR3  NTRK1  NTRK3  PDGFRA  PPARG  RAF1  RET  ROS1  AKT2  AR  BRCA1  BRCA2  CDKN2A  ERB84  ESR1  FGR  FLT3  JAK2  KRAS  MDM4  MET  MYB  MYBL1  NF1  NOTCH1  NOTCH4  NRG1  NTRK2  NUTM1  PDGFRB  PIK3CA  PRKACA  PRKACB  PTEN  RAD51B  RB1  RELA  RSPO2  RSPO3  TERT |
